# Beyond the Page: Solar Loading Thermographic Imaging and Predictive Modeling for Ancient Book Diagnostics—Preliminary Results

**DOI:** 10.3390/s26041358

**Published:** 2026-02-20

**Authors:** Elena Marini, Gilda Russo, Hai Zhang, Stefano Sfarra

**Affiliations:** 1 Department of Industrial and Information Engineering and Economics, University of L’Aquila, I-67100 L’Aquila, Italy; elena.marini@guest.univaq.it (E.M.); gilda.russo@guest.univaq.it (G.R.); 2 Centre for Composite Materials and Structures (CCMS), Harbin Institute of Technology, Harbin 150001, China; hai.zhang@hit.edu.cn

**Keywords:** solar loading thermography, ancient book, numerical simulation, multidimensional fast iterative filter, principal component thermography, robust principal component thermography, COMSOL Multiphysics^®^

## Abstract

**Highlights:**

**What are the main findings?**

Non-destructive techniques (NDTs) effectively detect sub-surface fabricated defects in an ancient book, with improved accuracy for certain material types.

- Advanced image processing: Multidimensional Fast Iterative Filtering (MdFIF), Principal Component Thermography (PCT), and Robust Principal Component Thermography (RPCT), combined with numerical simulations (COMSOL Multiphysics^®^), enhance diagnostic precision, especially for difficult to reveal hidden defects.

**What are the implications of the main findings?**

These methods significantly enhance the monitoring of the conservation of movable cultural heritage objects, enabling more accurate and timely interventions.

- The use of a numerical simulation model is particularly advantageous when dealing with movable artworks, especially valuable pieces that cannot be exposed to direct solar loading during testing.

**Abstract:**

This study investigates the application of NDTs for the detection of sub-surface defects in an ancient book, with the aim of improving conservation methods in the field of cultural heritage. A sequence of thermographic images in a solar loading thermography (SLT) scenario was acquired during a diagnostic campaign in Harbin, China, to identify four distinct fabricated dowels made of Wool, Rubber, Teflon^®^, and Synthetic material. The images were processed in two ways: the first combined advanced image-processing methods: pre-processing via MdFIF, post-processing, PCT and RPCT, applied both to the original sequence and to the MdFIF-filtered thermograms. The second approach employed numerical simulations in COMSOL Multiphysics^®^ to develop a predictive thermal model. The comparison of localized thermal anomalies obtained from the two approaches demonstrated the capability of NDTs to reliably reveal artificial defects, confirming their suitability for diagnostic conservation. Overall, the integration of advanced image processing with numerical simulation enhances diagnostic accuracy, particularly for subtle or low-contrast anomalies, thereby enabling more informed condition assessment and supporting rapid, targeted, and preventive conservation strategies.

## 1. Introduction

Ancient books represent a particularly complex category of movable cultural heritage objects, characterized by a stratified structure composed of heterogeneous organic materials such as paper, paperboard, textiles, adhesives, and coverings [1]. Over time, natural aging processes, environmental exposure, and handling can progressively alter their mechanical integrity and thermophysical properties, often leading to delamination, loss of cohesion between layers, and localized degradation phenomena. The diagnosis of such alterations is a crucial step for conservation planning; however, it is inherently challenging due to the fragility of the objects and the need to avoid invasive or destructive approaches.

Infrared thermography (IRT) is a widely adopted methodology across various fields, including medicine [2], architecture [3], engineering [4], and, more recently, the study and conservation of cultural heritage [5,6,7].

IRT has increasingly demonstrated its potential as a non-destructive testing technique for the investigation of cultural heritage artifacts. Its capability to detect subsurface features and material inhomogeneities based on thermal contrast makes it suitable for the assessment of layered systems, including books, manuscripts, and archival materials [8]. Among the different thermographic approaches, SLT is especially attractive for movable heritage objects, as it exploits natural solar radiation as a low-intensity, spatially distributed energy source, eliminating the need for artificial heating devices that could pose risks to delicate materials [9,10]. So, while artificial active thermography offers superior controllability in laboratory settings, this study advocates for an engineering-oriented in situ approach. Passive solar loading is utilized to address the diagnostic needs of heritage assets or artifacts located in some place where electrical infrastructure for active equipment is absent or impractical. This way, the risks associated with the use of active thermal imaging can be avoided.

Despite these advantages, the application of SLT to ancient books remains limited, mainly due to the intrinsic low thermal contrast of organic materials and the strong influence of environmental disturbances, such as non-uniform illumination, ambient temperature fluctuations, and long acquisition times. These factors often mask weak thermal signatures associated with subsurface defects or material discontinuities, making raw thermographic data difficult to interpret reliably. Consequently, advanced signal and image processing techniques are required to enhance defect visibility and to reduce the impact of noise and artefacts.

In recent years, several post-processing methodologies have been introduced to improve defect detectability in thermographic sequences, including PCT and its robust variant, RPCT. These techniques exploit statistical decomposition to separate meaningful thermal patterns from background effects. However, their effectiveness strongly depends on the quality of the input data. For this reason, pre-processing strategies aimed at signal decomposition and noise attenuation, such as MdFIF [11,12,13], have emerged as powerful tools to enhance the information content of thermographic sequences prior to statistical analysis [2].

Parallel to experimental investigations, numerical simulations have become an increasingly valuable support for thermographic diagnostics. Predictive thermal models, implemented through finite-element software such as COMSOL Multiphysics^®^, allow the simulation of heat transfer phenomena under controlled boundary conditions, providing insight into the thermal behavior of complex, multilayered objects. In the context of cultural heritage, numerical modeling is particularly advantageous when experimental conditions cannot be freely modified, or when direct exposure to solar loading is not feasible for valuable artifacts. Moreover, simulations enable a physical interpretation of experimentally observed thermal anomalies by linking them to material properties, geometry, and energy transfer mechanisms [9,10].

Numerical simulations performed with modeling software, in this specific case COMSOL Multiphysics^®^, may present limitations in the completeness of the geometric model representation, particularly for complex objects—such as historical hardbacks—due to the following reasons: (1) lack of detailed geometric information of the real artifact; (2) excessive representational complexity which does not justify an increase in the quality of the numerical results; (3) high computational cost related to mesh thickening, replicating complex geometries, which do not lead to substantial variations in the results obtained compared to more essential models. Conversely, it is always possible to introduce the highest degree of complexity in terms of material properties, thermophysical properties, and representations of multiphysics scenarios. In fact, starting from a geometry with a number of known nodal elements and degrees of freedom (GdL), the introduction of isotropic or transversely isotropic, orthotropic and anisotropic materials is responsible for the only increase (proportional to the required complexity) in calculation time but in any case, for negligible values. What heavily modifies processing times, however, is the introduction of coupled multiphysics. In this specific case, natural convection affects the effects of time-varying air flow as well as thermohygrometric variations. The aim of this model, which focuses on a qualitative and comparative analysis of experimental thermographic results, was to support the physical understanding of the specific thermophysical model by evaluating the response of the materials by establishing a mutual interaction between the elements constituting the ancient book. In the model under analysis, there are only modest limitations in terms of coupled multiphysics [14].

Within this framework, the present study proposes an integrated diagnostic approach that combines SLT, advanced pre- and post-processing techniques, and numerical simulation for the investigation of an ancient book. A thermographic sequence acquired under solar loading conditions during a diagnostic campaign conducted in Harbin (China) was processed using MdFIF as a pre-processing step, followed by PCT and RPCT applied to both the original and filtered sequences. In parallel, a detailed three-dimensional numerical model of the book and its experimental setup was developed in COMSOL Multiphysics^®^, incorporating realistic material properties, geometry, radiative exchange, and natural convection effects.

The main objective of this work is twofold: (i) to evaluate the effectiveness of combined MdFIF–PCT–RPCT processing in enhancing the detectability of subsurface fabricated defects made of different materials within an ancient book, and (ii) to compare experimental thermographic outcomes with numerical predictions in terms of thermal fields and temperature gradients. By correlating experimental defects-related thermal signatures with simulated thermal responses, the study aims to assess the reliability and complementarity of the two approaches, ultimately contributing to the development of robust, non-invasive diagnostic protocols for the conservation of fragile book heritage.

## 2. Materials and Methods

In this paper, the effects of temperature on the identification of defects in an ancient book are investigated through both an experimental thermographic campaign and numerical simulations performed using COMSOL Multiphysics^®^ v. 6.3. The following section describes the analyzed sample, the experimental setup, the applied thermal image-processing techniques, and the development of the predictive numerical model.

### 2.1. Sample: Ancient Book

**Figure 1:**
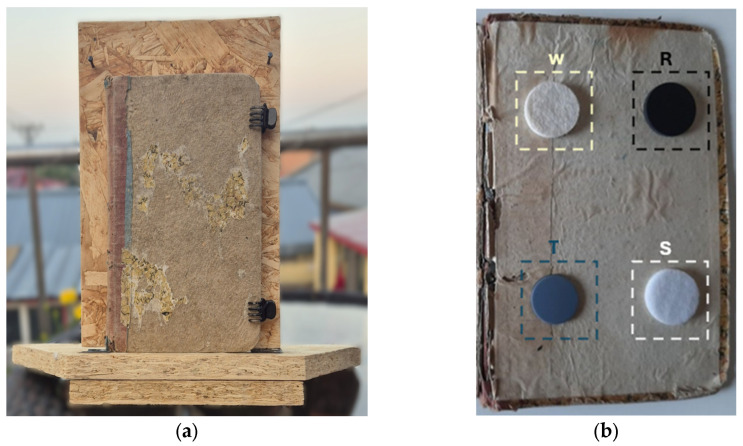
(**a**) Analyzed sample showing signs of degradation, including delamination of the front cover; (**b**) The four defects inserted inside the book: wool (W), rubber (R), Teflon (T), and synthetic material (S).

The sample examined (Figure 1a) is a 19th-century book (18 × 12 × 1.5 cm), published by Luigi Beuf Libraio, selected from a private collection due to its historical value and its state of preservation. The book exhibits clear signs of degradation, including extensive delamination of the front cover, which highlights the natural deterioration of the material over time. This type of wear serves as evidence of the long period of aging and the environmental conditions to which the volume has been exposed over the decades. As shown in Figure 1, within the book, behind the front cover, four circular dowels made from different materials were inserted, positioned near the corners of the book. The selected materials are arranged as follows: wool (W) in the upper-left quadrant, rubber (R) in the upper-right, Teflon^®^ (T) in the lower-left, and a synthetic material (S) in the lower-right quadrant (Figure 1b). The choice of these materials was driven by the intention to study the different responses of each material to infrared thermography (IRT), with the aim of assessing their detectability or lack thereof. The selection of rubber and synthetic inserts was intended to reproduce low-contrast subsurface inhomogeneities representative of polymer-based or compliant materials that may be present in historical books as a consequence of past restoration practices, conservation accessories, or modern handling. From a thermographic perspective, these materials simulate challenging defects characterized by thermal diffusivity values closer to that of the paperboard host, allowing the robustness of the proposed MdFIF–PCT/RPCT processing framework to be evaluated under realistic solar loading conditions. Furthermore, the book was kept closed using clips, preventing the entry of air into the volume, thereby reducing the risk of altering the acquisition conditions and ensuring the reliability of the results.

### 2.2. Numerical Model with COMSOL^®^

**Figure 2:**
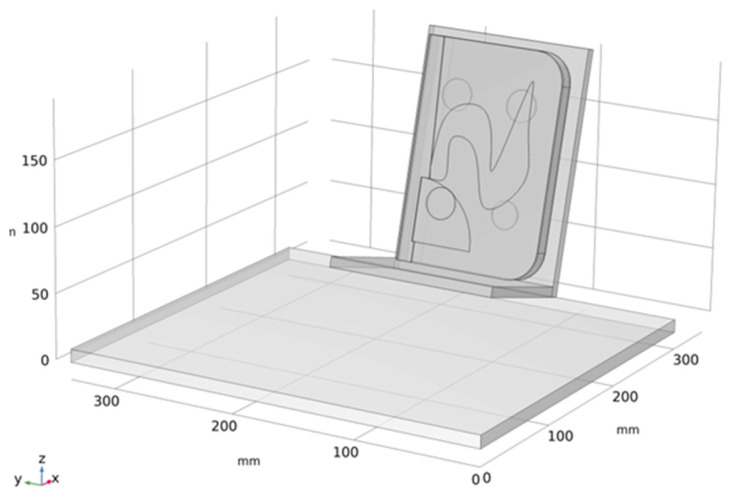
Three-dimensional orientation of the numerical model under solar loading, configured to match the sample inclination and positioning during testing.

A numerical model is a computational representation of a physical system that enables the simulation of its behavior under prescribed conditions, allowing physical phenomena to be predicted and analyzed without extensive experimental testing. In this study, the numerical model was developed in COMSOL Multiphysics^®^ based on a detailed examination of the real book, accounting for both thermophysical properties and a faithful geometric replication of the main structural features. A 3D CAD model of the book was created in AutoCAD^®^2025 (AutoDESK) and then imported into COMSOL through LiveLink™ for AutoCAD^®^. LiveLink™ ensured an accurate transfer of the geometry, including the conversion of local CAD coordinates into COMSOL global coordinates, thereby preserving the correct alignment with the experimental configuration. The model was oriented to reproduce the real inspection scenario under solar loading conditions, as shown in Figure 2.

As described above, the book model includes multiple materials, namely wool, Teflon^®^, synthetic insert, and rubber, as well as paper/cellulose for the text-block, cotton for the spine, paperboard for the cover structure, and polyvinyl acetate for the adhesive exposed in the decorticated areas. The thermophysical properties assigned to each material were sourced from the literature or obtained from the COMSOL material library and are summarized in Table 1.

**Figure 3:**
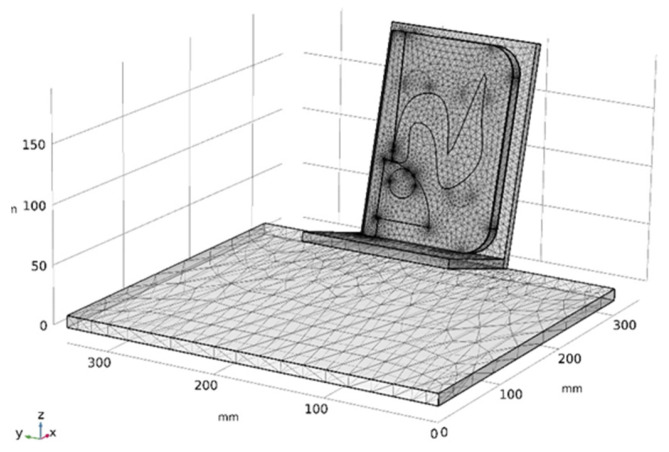
Tetrahedral mesh of the entire model.

OSB is a wooden essence made up of undirected chips, recovered from processing waste, and glued with the aid of mechanical pressure. This turns out to be a typical material for making mock-ups for ease of processing and at low cost. The numerical simulations were performed using the Heat Transfer with Surface-to-Surface Radiation [22] physics interface. The governing heat transfer equation is given by Equation (1):

(1) $$\rho Cp\frac{\partial T}{\partial t}+\rho Cp\;u\cdot \nabla T=\nabla \cdot (k\nabla T)+q$$

where *ρ* is the density [kg/m^3^], *Cp* is the specific heat at constant pressure [J/kg·K], *k* is the thermal conductivity tensor [W/m·K], *T* is the temperature [K], and *q* is the heat flux vector field according to Fourier’s law of heat conduction. Emissivity boundary conditions were applied to all model surfaces, and the initial temperature was set to 293.66 K, corresponding to the temperature recorded during the experimental data acquisition. For the OSB panels supporting the book, a thermal boundary condition Equation (2):

(2) −n· (−k∇T)=0

was imposed on the surfaces not directly exposed to solar radiation, ensuring thermal equilibrium with the surrounding environment at the initial time step. Radiative heat transfer was accounted for on all surfaces, including the OSB panels; therefore, albedo and energy absorption effects were included through the appropriate boundary-condition settings. Natural convection was also considered by applying heat flux [17] conditions to all surfaces subjected to direct solar radiation. The convective heat flux was modeled as Equation (3):

−q_0_ = *h*(T_ext_ − T) (3)

where *q*_0_ is the convective heat flux [W/m^2^], *h* is the heat transfer coefficient [W/m^2^K], and *T_ext_* is the external temperature [K], representing the ambient environment temperature. The thermal excitation in the experimental setup was provided by solar radiation. The solar spectrum was modeled as that of a black body at 5780 K [23] through Planck’s law. Solar irradiance values were obtained from literature and used to compute the average solar irradiance, *Is*, accounting for the Earth–Sun distance and then implemented in COMSOL Multiphysics^®^ as the thermal load input [24,25]. The model was discretized using a tetrahedral mesh (Figure 3) and solved on a Windows 11 system with an Intel64 CPU and 31.73 GB of RAM. A time-dependent solver was adopted with a 10-min frame rate, simulating the interval from 6:00 A.M. to 7:00 P.M. to match the duration of the experimental acquisition. It is also important to clarify that, within COMSOL Multiphysics^®^, in addition to defining thermophysical and structural parameters, it is possible to explicitly specify the crystalline state of a material (i.e., isotropic, transversely isotropic, orthotropic, or fully anisotropic). This can be implemented through two different approaches.

The first approach applies to materials available in the integrated COMSOL material library. After selecting the appropriate “physics” or “multiphysics” interface, the modeler assigns a material to a given geometric domain through the “material model” settings. Within this framework, different constitutive behaviors can be selected, such as linear elastic material, nonlinear elastic material, hyperelastic material, rigid material, shape memory alloy, and others. The choice of the constitutive model is domain-specific and therefore directly associated with each material composing the numerical geometry.

The second approach applies to materials not included in the integrated library. In this case, the workflow is conceptually identical; however, all required material parameters must be explicitly defined by the modeler in the “parameters” section of the software. For instance, when modeling an anisotropic material, the complete elasticity matrix must be specified. All tensor components required by the governing equations must therefore be individually introduced.

In the present study, all parameters necessary to correctly describe the crystalline state of each material included in the numerical model were explicitly defined, ensuring full consistency between the adopted material formulation and the intended physical representation. Overall, the simulation provides insights into the book about thermal response under realistic solar loading conditions, supporting the interpretation of material behavior and the identification of defect-related signatures.

For clarity, the procedure adopted to identify defect-related results is defined consistently for both the numerical approaches and the experimental. In the proposed analytical workflow, localized thermal anomalies (defect-related thermal signatures) are defined as spatially localized, high-contrast features appearing within selected EOFs produced by PCT/RPCT (applied to either the raw or MdFIF-filtered sequence). These features are identified inside defect-specific Regions of Interest (ROIs) by maximizing a normalized contrast index with respect to a sound background region.

### 2.3. Experimental Set up and Data Acquisition

This study employed SLT, a passive technique that utilizes solar heat as an external energy source for thermal measurements. The method facilitates the acquisition of thermographic images without the need for artificial energy sources, thereby minimizing any potential modifications to the artifact’s material composition.

**Figure 4:**
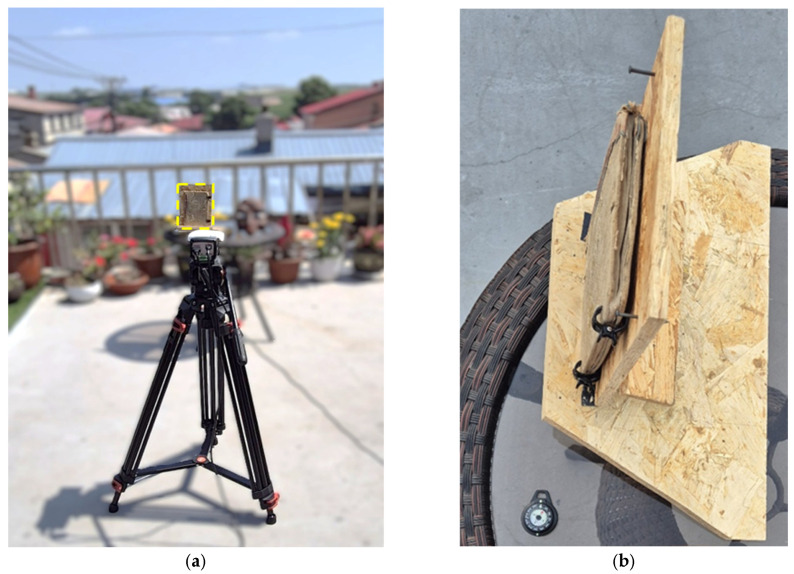

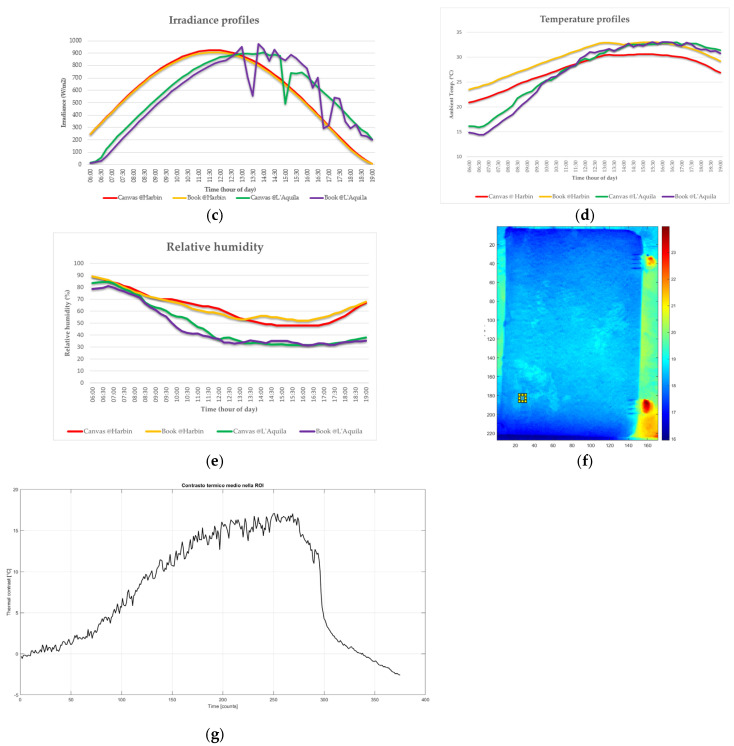
(**a**) The setup for thermographic data acquisition, featuring the thermal camera positioned on a tripod, with the sample placed on a stable table; (**b**) Close-up of the book secured in place with clamps; (**c**) Measured solar irradiance as a function of time during the thermographic acquisition (22 July 2025, Harbin, China). The data illustrate the non-stationary nature of the solar thermal forcing acting on the sample during the 13-h inspection; (**d**) Ambient temperature profiles recorded during the experimental campaign as a function of time of day. Measurements were acquired at 15-min intervals, highlighting the diurnal thermal cycle and the gradual increase in temperature associated with solar loading conditions; (**e**) Relative humidity profiles measured during the experimental campaign as a function of time of day. Data recorded at 15-min intervals show an inverse trend with respect to ambient temperature, reflecting typical atmospheric behavior under natural solar loading conditions. (**f**–**g**) Temporal evolution of the surface temperature of the book recorded during the solar loading acquisition, confirming the limited thermal excursion and the conservative nature of the applied thermal excitation. Readers should refer to the box present in f).

The diagnostic campaign presented in this study forms part of a comprehensive two-year research program (sCHans), which also included previous investigations conducted in 2024 in L’Aquila, Italy. The final data acquisition was carried out on 22 July 2025, in Harbin, China, at a site located at 45.68706° N and 126.734252° E. To ensure consistency and methodological continuity in the experimental setup, following the protocol described by Ding et al., 2025 [26], the acquisition session lasted a total of thirteen hours, beginning at 6:00 A.M. and concluding at 7:00 P.M. Due to institutional regulations of the Harbin Institute of Technology (HIT), the explicit model name and full technical specifications of the thermal camera cannot be disclosed. Nevertheless, all relevant non-sensitive characteristics required for data interpretation and reproducibility, including sensor type (LWIR microbolometer), spatial resolution (640 × 480 pixels), acquisition duration, and safety monitoring procedures, are provided (Figure 4a). This instrument was selected for its high precision and proven effectiveness in detecting defects in cultural heritage artifacts. To ensure the safety of the historical artifact, the surface temperature was continuously monitored throughout the 13-h acquisition. A high-temperature alarm was set on the thermal camera to verify that the sample remained within safe thermal preservation thresholds at all times. The sample was placed on a stable wooden tripod to ensure a secure and stationary position throughout the entire acquisition process (Figure 4b). Furthermore, solar irradiance, relative humidity, and ambient temperature were recorded at 15-min intervals. Solar irradiance, temperature and relative humidity were monitored throughout the entire acquisition period and recorded at 15-min intervals. Figure 4c reports the measured solar irradiance as a function of time during the experimental campaign, highlighting the non-stationary nature of the thermal excitation due to cloud cover variability and atmospheric conditions. These data provide the environmental context of the solar loading conditions applied to the sample.

In addition, the temporal evolution of ambient temperature and relative humidity was continuously monitored to provide a complete characterization of the environmental boundary conditions acting on the sample. Figure 4d shows the ambient temperature profiles measured during the inspection day, highlighting the typical diurnal thermal cycle with a gradual increase from early morning to midday followed by a stabilization and a slight decrease in the late afternoon. Figure 4e reports the corresponding relative humidity trends, which exhibit an inverse behavior with respect to temperature, characterized by higher values in the early morning and lower values during peak solar exposure. These environmental measurements confirm the non-stationary nature of the thermal excitation and provide essential contextual information for interpreting the thermographic response under natural solar loading conditions.

Figure 4f–g reports the temporal evolution of the surface temperature recorded during the 13-h solar loading acquisition. The profile confirms that the thermal excitation remained within a narrow and safe range throughout the inspection, while exhibiting the slow, non-stationary behavior characteristic of natural solar loading.

Wind speed and wind direction were also recorded as spot measurements during the acquisition to document the outdoor convective conditions affecting surface heat exchange (Table 2) [27].

### 2.4. Analytical Methods

Following the acquisition of the sequence in SLT mode, the study utilizes advanced pre-processing techniques, specifically MdFIF, combined with post-processing methods such as PCT and RPCT. The application of these techniques to thermal image sequences is aimed at enhancing the detectability of defect-related thermal signatures under natural solar loading conditions, where the thermal contrast is typically low and the measurements are affected by environmental variability. In this framework, pre-processing is primarily intended to attenuate disturbances and improve the signal quality prior to any statistical decomposition, whereas post-processing is used to extract interpretable components and emphasize spatial patterns potentially associated with subsurface discontinuities. Overall, this combined strategy supports a more reliable interpretation of the thermographic sequence by reducing the occurrence of false positives and limiting the risk of misleading artifacts introduced by noise, non-uniform heating, or temporal fluctuations. In the subsequent sections, the authors provide a detailed exposition of the adopted techniques and the rationale for their application to both the raw and the pre-filtered sequences.

#### 2.4.1. Multidimensional Fast Iterative Filtering

As previously mentioned, the acquired thermal images can be influenced by several environmental and operational factors that compromise their interpretation and clarity. Typical disturbances include gradual temperature drifts during the day, local variations in solar irradiance, wind-induced convection changes, and measurement noise intrinsic to the camera system. In such conditions, the thermal response associated with subsurface defects may be partially masked or mixed with unrelated fluctuations, making it necessary to introduce a dedicated pre-processing stage to reduce disturbances while preserving meaningful information.

To mitigate these effects, the present study adopts MdFIF on the original thermographic sequence [11,12,13]. While Ding et al., 2025 [26] applied Multivariate Fast Iterative Filtering (MvFIF), MdFIF is here preferred because it directly exploits the multidimensional nature of the dataset by jointly leveraging spatial and temporal information embedded in the image sequence. MdFIF is an advanced data-driven method based on iterative filtering and signal decomposition: it separates the original sequence into components characterized by different spatial–temporal scales, allowing the attenuation of high-frequency noise contributions and other undesired disturbances. In practical terms, the pre-filtered sequence obtained after MdFIF processing exhibits reduced noise content and improved spatial coherence, which is beneficial for subsequent statistical analysis.

The proposed approach is particularly suitable for dynamic sequences such as thermographic acquisitions, because it preserves relevant details while attenuating disturbances without compromising spatial or temporal resolution. This aspect is critical for solar loading thermography, where the signal of interest is not a single transient pulse but a slowly varying thermal evolution driven by natural excitation. Moreover, MdFIF is computationally efficient and can handle large datasets rapidly, making it well suited for applications requiring intensive thermal image processing and enabling its integration into routine workflows.

It is pertinent to highlight why MdFIF is preferred over traditional multi-scale decomposition methods, such as the Wavelet Transform, in the specific context of SLT. Wavelet-based approaches rely on fixed basis functions (mother wavelets) to decompose signals. When applied to the highly non-stationary and stochastic thermal drifts caused by variable solar loading (e.g., shifting cloud cover and changing solar angles), these fixed bases often fail to match the irregular shape of the environmental trends. This leads to basis mismatch and subsequent spectral leakage, where the energy of the solar drift is not effectively confined to low-frequency components but spreads into the defect-related frequency bands, creating artifacts. In contrast, MdFIF employs an adaptive, data-driven decomposition process that does not presume a predefined functional shape. By sifting the signal based on its local extrema rather than a fixed global basis, MdFIF can accurately track and separate the complex, non-linear trends of solar forcing from the subtle, transient thermal signatures of subsurface defects, thereby theoretically justifying its selection over fixed-basis methods for this application.

#### 2.4.2. PCT and RPCT

A second crucial step in the elaboration of thermal images is post-processing, which involves the use of statistical techniques such as PCT and RPCT for dimensionality reduction and data interpretation [28,29,30,31]. In this study, these methods were applied both to the original sequence and to the sequence previously processed with MdFIF. This dual application was adopted to systematically evaluate whether an initial pre-filtering stage can improve the performance of the statistical decomposition and facilitate defect identification in challenging measurement conditions.

PCT is a well-established technique for dimensionality reduction. It enables the transformation of the thermographic dataset into a set of orthogonal components that capture the dominant variance modes of the sequence. In the context of infrared thermography, these components (often interpreted as Empirical Orthogonal Functions, EOFs) can reveal spatial patterns that may correspond to different physical contributions, such as global heating trends, non-uniform illumination, environmental disturbances, and, when present, localized anomalies related to defects. By concentrating the relevant information into a limited number of components, PCT improves the interpretability of the data and facilitates the identification of features of interest that may be difficult to observe in the raw thermograms.

From a mathematical perspective, PCT is implemented by reshaping the thermographic sequence into a two-dimensional data matrix X ∈ ℝ^(N × M), where N represents the number of spatial pixels and M the number of temporal frames. The covariance matrix C is defined as:

C = (1/M)···X···Xᵀ(4)

The associated eigenvalue problem:

C···v_i = λ_i···v_i(5)

is then solved, where v_i are the eigenvectors, commonly referred to as Empirical Orthogonal Functions (EOFs), and λ_i are the corresponding eigenvalues. Each EOF represents a spatial thermal pattern, while the eigenvalues quantify its contribution to the total variance of the dataset. In infrared thermography, lower-order EOFs are generally dominated by global heating trends and environmental effects, whereas higher-order EOFs may reveal localized thermal anomalies associated with subsurface defects. Consequently, the selection of relevant EOFs is data-driven and guided by physical interpretability rather than by fixed numerical thresholds.

However, PCT is known to be sensitive to noise and defect indication, because variance-based decomposition can be strongly influenced by anomalous values or residual disturbances in the dataset. When the thermographic sequence is contaminated by environmental fluctuations or measurement noise, the extracted components may emphasize unwanted contributions, potentially reduce the visibility of defect-related patterns or generate spurious features that can be misinterpreted. To address this limitation, RPCT was also adopted.

RPCT is formulated by decomposing the thermographic data matrix X into the sum of a low-rank component L and a sparse component S, according to:

X = L + S(6)

This decomposition is obtained by solving the optimization problem:

min_{L,S} ||L||_* + λ ||S||_1_ (7)

subject to X = L + S, where ||L||_* denotes the nuclear norm, promoting low-rank structures associated with global thermal behavior, and ||S||_1_ denotes the ℓ_1_-norm, which promotes sparsity and isolates localized thermal anomalies. The regularization parameter λ balances the two terms and is selected following standard recommendations in the literature. This formulation allows RPCT to effectively separate defect-related thermal signatures from non-stationary environmental disturbances typical of solar loading thermography.

Unlike standard PCT, RPCT is specifically designed to be more robust to noise and outliers: it applies a methodology that more effectively separates coherent low-rank structures (i.e., physically meaningful trends) from sparse or incoherent perturbations (i.e., residual noise, localized artifacts, or anomalous variations). As a result, RPCT can preserve relevant spatial details and enhance defect detectability even when the data are affected by disturbances.

In summary, while PCT is suitable for analyzing relatively clean thermographic datasets and provides an efficient first-level decomposition of the thermal evolution, RPCT proves advantageous in more challenging conditions by improving the stability of the extracted components, reducing the likelihood of false positives, and increasing the reliability of defect identification. For these reasons, both techniques were considered in the present study and applied to the raw and MdFIF-filtered sequences to assess their complementarity and to support a consistent interpretation of the results.

In the numerical approach, the corresponding defect-related thermal signatures are represented by localized extrema (hotspots) in the simulated surface temperature variation (ΔT) and, more robustly, in the temperature gradient magnitude (|∇T|). These quantities are evaluated over the same defect ROIs and at time instants matching the experimental acquisition (or corresponding to maximum simulated contrast), thus enabling a direct cross-comparison between experimental EOF-based anomalies and model-predicted thermal signatures. The authors specify that generative artificial intelligence (GenAI) tools were used exclusively for English language editing of the manuscript, including grammar, spelling, and stylistic refinement. No GenAI was employed in the study of design, data analysis, interpretation of results, or preparation of figures or scientific content.

## 3. Results

This section is divided into two subheadings to help the reader follow the discussion of the results.

### 3.1. COMSOL-Based Thermal Field and Gradient Analysis

**Figure 5:**
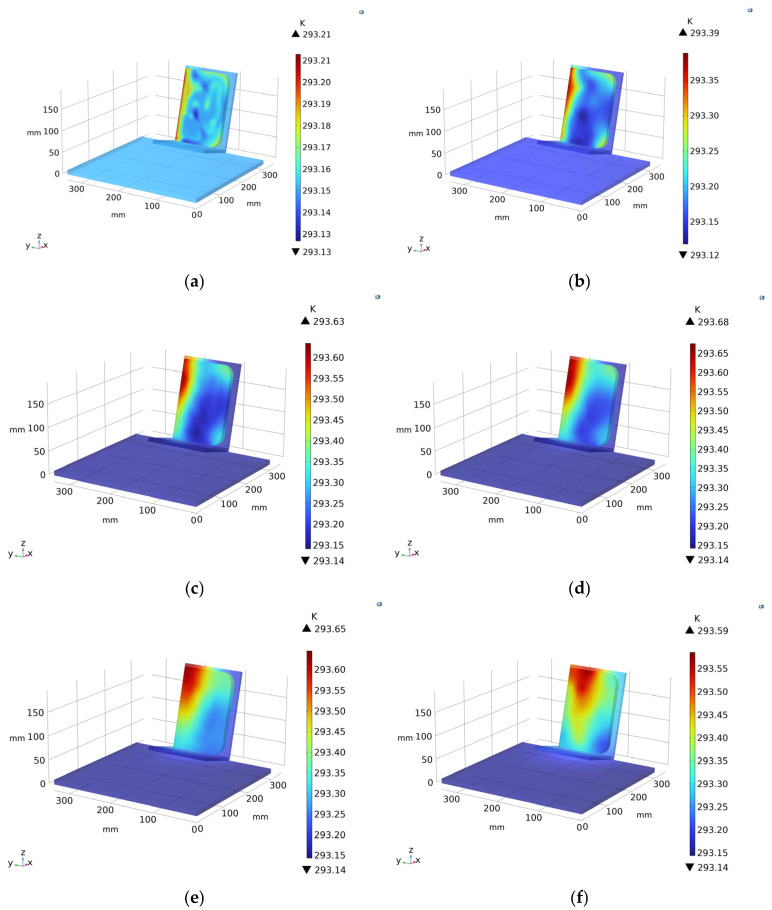

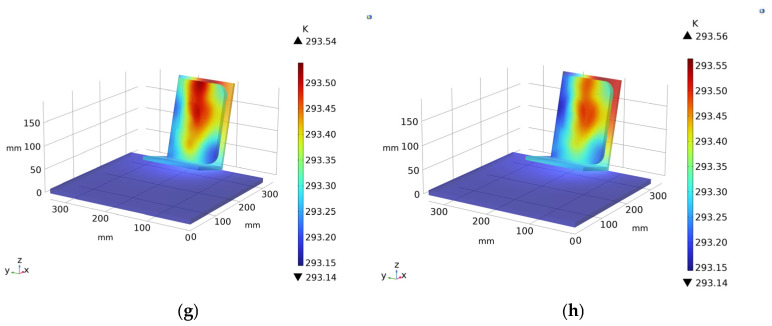
Temperature scenarios reconstructed according to the experimental setup: (**a**) temperature distribution at 6:10 A.M.; (**b**) at 7:00 A.M.; (**c**) at 10:00 A.M.; (**d**) at 12:00 A.M.; (**e**) at 2:00 P.M.; (**f**) at 4:00 P.M.; (**g**) at 6:00 P.M.; and (**h**) at 7:00 P.M.

The numerical simulation carried out in COMSOL Multiphysics^®^ reproduced the thermal response of the book cover under natural solar loading at selected time intervals spanning the entire acquisition day, from early morning (06:10 A.M.) to late afternoon (07:00 P.M.). The choice of analysis time of approximately 13 h is linked to the solar thermal load, which typically stresses paper volumes. Unless the ancient books are properly preserved, they are thermally stressed through solar loading, which in any case does not harm the volumes. If we wanted to reduce the time to investigate an ancient book, always making sure not to use heat sources that could damage the volume, we would have to use lighting fixtures in the visible or infrared spectrum by acting on the choice of appropriate power and frequency. This is to quickly assess the possible presence of defects, modulate the frequency, and to quickly investigate the possible depths of the artifact. The computed surface temperature fields (Figure 5a–h) show very limited absolute thermal excursions throughout the day, with temperature ranges of approximately 0.08 K at 06:10 A.M., increasing progressively to values between about 0.40 K and 0.54 K during the central hours (10:00 A.M.–04:00 P.M.), and remaining within a comparable range during the late afternoon (06:00 P.M.–07:00 P.M.). These small temperature variations confirm the strongly insulating behavior of the layered book structure and the smoothing effect of the decorative surface layer, which tends to homogenize the thermal field even under peak irradiance conditions.

Despite the low thermal contrast, the simulated temperature maps clearly reveal spatially coherent patterns associated with subsurface material heterogeneities. Areas affected by surface decortication, modelled as thin residual layers of polyvinyl acetate, systematically exhibit a thermal response distinct from intact regions at all simulated times (Figure 5). These zones act as preferential pathways for thermal contrast development, enabling the partial visibility of underlying inserts. Among the simulated materials, wool and Teflon^®^ dowels produce the most detectable signatures, while rubber and synthetic dowels remain weakly expressed over the entire temporal sequence. The Teflon^®^ insert is particularly enhanced when located beneath decorticated areas, as observed during the mid-day and afternoon simulations (12:00 A.M.–04:00 P.M.), indicating a combined effect of surface condition and subsurface material properties.

**Figure 6:**
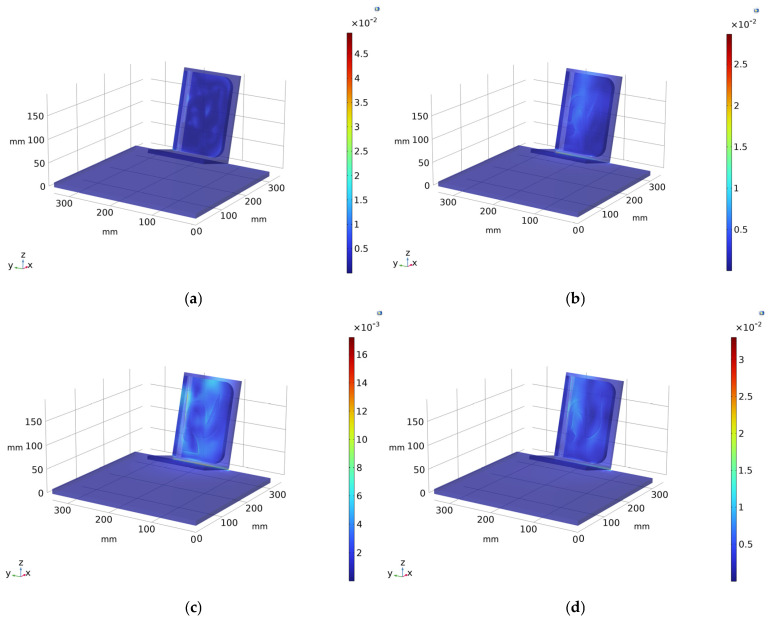
Temperature gradient scenarios [K/mm]: (**a**) temperature gradient at 6:10 A.M.; (**b**) at 2:00 P.M.; (**c**) at 4:00 P.M.; and (**d**) at 7:00 P.M.

Further insight is provided by the temperature gradient maps (Figure 6a–d), which highlight localized variations in heat flux rather than absolute temperature. At early hours (06:10 A.M.), the gradient distribution appears relatively uniform, with only subtle anomalies corresponding to decorticated regions. As solar loading increases (02:00 P.M.–04:00 P.M.), sharper gradient contrasts emerge, especially at the interface between decorticated areas and Teflon^®^ inserts, whereas wool-related features remain detectable even without surface degradation. At 07:00 P.M., although solar irradiance is reduced, the contrast between decorticated regions and Teflon^®^ remains appreciable, confirming the persistence of thermally driven material-dependent behavior.

The three-dimensional temperature and gradient distributions shown in the COMSOL renderings (Figure 5 and Figure 6), corresponding to the images provided here, further emphasize the spatial coherence of these effects and the role of geometry and boundary conditions in governing heat diffusion. Overall, the numerical results indicate that detectability is not controlled solely by thermal conductivity, but is primarily governed by differences in thermal diffusivity, influenced by material density and heat capacity. The simulation thus provides a robust physical framework for interpreting experimental thermographic observations and for understanding the role of surface degradation in enhancing subsurface defect visibility.

### 3.2. Identification of Fabricated Defects in Thermal Images

**Figure 7:**
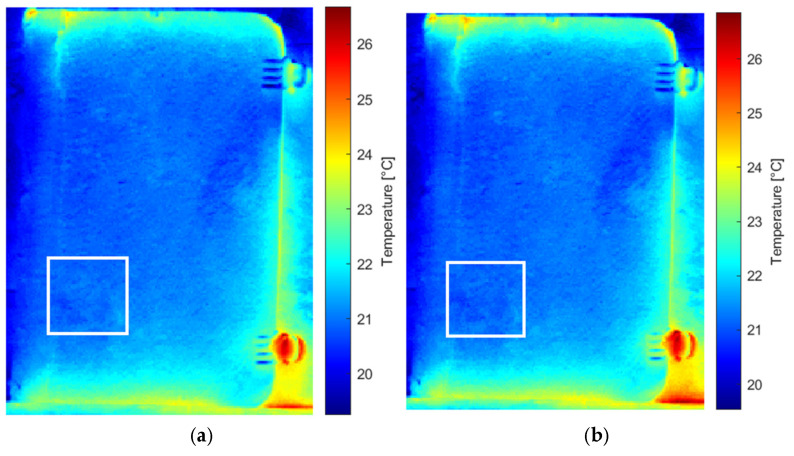
Original thermal sequence: (**a**) 75th and (**b**) 76th images indicating the Teflon defect, outlined by white square markers.

**Figure 8:**
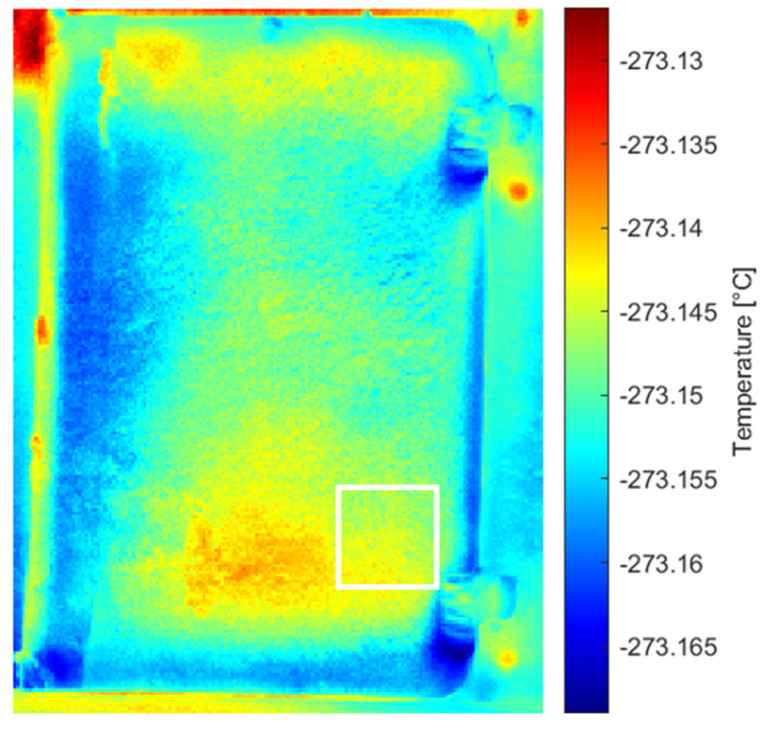
RPCT results for the original sequence: the 9th EOF revealing the synthetic defect, outlined by white square markers.

Analysis of the original thermal image sequence revealed the Teflon defect in the 75th and 76th images (Figure 7). The images were then examined following processing with different methods, including pre-processing (MdFIF) and statistical post-processing techniques (PCT and RPCT). When PCT was applied to the original sequence, no significant EOFs were detected. More notable results were obtained when analyzing the original sequence processed with RPCT, where the 9th EOF clearly highlights the circular trace associated with the synthetic material defect (Figure 8).

**Figure 9:**
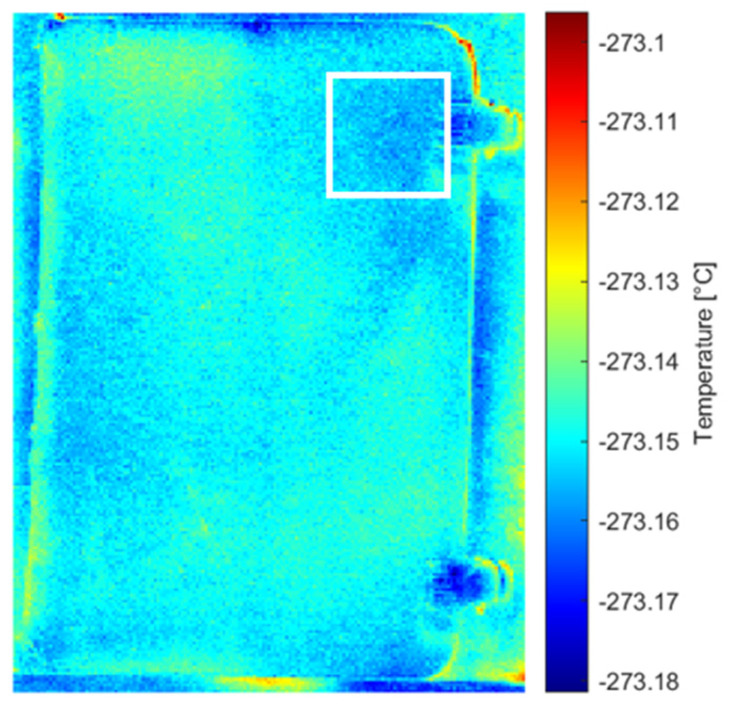
PCT applied to the MdFIF-filtered sequence: the 26th EOF reveals the trace of the rubber defect, outlined by white square markers.

**Figure 10:**
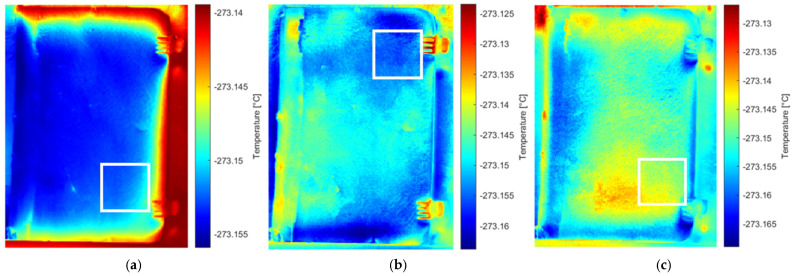
RPCT applied to the MdFIF-filtered sequence: (**a**) 2nd and (**c**) 9th EOFs reveal the traces of the synthetic material defects, while (**b**) the 8th EOF highlights the rubber defect.

In the thermograms derived from pre-filtered sequences and post-processed using PCT, the rubber defect was the most prominently visible (Figure 9). Finally, the sequence pre-processed with MdFIF and subsequently analyzed with RPCT was considered. The 2nd, 8th, and 9th EOFs distinctly reveal the defects associated with both rubber and synthetic material (Figure 10).

Finally, for this study, due to the non-stationary nature of solar loading (characterized by stochastic wind and cloud effects), traditional Signal-to-Noise Ratio (SNR) calculations yield statistically unstable results; therefore, this study focuses on the qualitative extraction of structured features. Furthermore, while geometrically regular artificial defects were used here as a ground truth for algorithmic calibration, the underlying thermal detection mechanism remains applicable to natural defects.

## 4. Discussion

The thermographic dataset acquired under natural solar loading conditions represents a particularly challenging inspection scenario due to the intrinsically low thermal contrasts of paper-based materials, the absence of controlled external excitation, and the influence of environmental variability over long acquisition times. Under these conditions, both experimental observations and numerical simulations highlight that defect detectability cannot be interpreted solely in terms of absolute surface temperature differences.

In transient infrared thermography, defect detectability is governed by the dynamic heat transfer behavior of materials rather than by absolute temperature values. A key parameter controlling this behavior is thermal diffusivity, which describes the rate at which a material responds to a thermal perturbation and redistributes heat over time. In the present study, the four fabricated defects (wool, rubber, synthetic material, and Teflon^®^) are embedded within a paperboard matrix; consequently, defect visibility is primarily controlled by the relative contrast in thermal diffusivity between each insert and the surrounding paperboard, rather than by their absolute thermophysical properties.

Paperboard, which constitutes the host material for all inserts, exhibits a thermal diffusivity of approximately 5.7 × 10^−8^ m^2^/s Wool and Teflon^®^ show diffusivity values that differ from this reference by several orders of magnitude, whereas rubber and the synthetic dowel display diffusivity values of the same order of magnitude as paperboard (see Table 1). This strong heterogeneity directly governs the transient thermal response and, in turn, the detectability of the defects.

Wool, characterized by extremely low density and a thermal diffusivity of about 2.6 × 10^−4^ m^2^/s, exhibits a slow but persistent thermal response in the COMSOL-based numerical simulations. A deeper understanding of this behavior requires moving beyond thermal diffusivity alone and explicitly considering the role of volumetric heat capacity. Thermal diffusivity α is defined as: α = k/ρc_p_ where k is the thermal conductivity, ρ the density, and c_p_ the specific heat at constant pressure. While diffusivity governs the rate at which a material reacts to thermal perturbations and redistributes heat, the term ρcp, the volumetric heat capacity, governs its energy storage capability. For the materials considered here, the volumetric heat capacity of Wool is: ρc_p_ = 22 × 8.24 = 181 J/m^3^K, whereas for Rubber it is: ρc_p_ = 1100 × 1000 = 1.1 × 106 J/m^3^K. Therefore, the energy storage capacity of Rubber is approximately 6000 times greater than that of Wool, while their thermal conductivities differ by only about one order of magnitude. Although Wool exhibits a very high thermal diffusivity—primarily due to its extremely low volumetric heat capacity—its ability to store thermal energy is extremely limited. Under quasi-static solar loading, characterized by very low excitation frequency and slow thermal forcing, this distinction becomes crucial. The solar input evolves slowly over time, meaning that the system response is dominated by energy storage effects rather than rapid transient propagation. In the layered configuration analyzed here, the overlying paperboard and polyvinyl acetate layers possess relatively high volumetric heat capacity and act as a thermal buffer. Their thermal inertia promotes homogenization of the surface temperature field. Because Wool stores very little energy, it does not introduce a significant local energy imbalance relative to the surrounding layers. As a consequence, the surface thermal field above the Wool insert remains spatially regular and lacks sufficiently pronounced localized gradients for experimental detectability. This explains why, despite its large diffusivity contrast, the corresponding defect does not emerge as a distinct feature in the experimental thermographic sequences under solar loading conditions. The masking effect results from the combined influence of low excitation intensity, surface thermal homogenization, and limited sensitivity to deep or weakly contrasted anomalies.

Teflon^®^, with a diffusivity of approximately 2.5 × 10^−5^ m^2^/s, also presents a marked contrast relative to the paperboard matrix. However, due to its higher diffusivity, the associated thermal signature is more transient and becomes clearly visible only within specific temporal windows. This effect is further enhanced by the specific configuration of the specimen, in which the insert is located beneath a decorticated area; the reduced thickness of the surface layer limits thermal homogenization and facilitates the transfer of diffusivity contrast to the surface.

Conversely, rubber and synthetic dowels exhibit thermal diffusivity values of approximately 1.4 × 10^−7^ m^2^/s and 1.6 × 10^−8^ m^2^/s, respectively, which are comparable to that of paperboard. As a result, their transient thermal response closely follows that of the host material, preventing the development of significant temperature or gradient discontinuities at the surface. In addition, the relatively high thermal inertia of the synthetic dowel further smooths its response over time, leading to a weak or negligible thermal contrast under solar loading conditions.

These thermophysical considerations are fully consistent with the results obtained from the statistical analysis of the thermographic sequence. In the original sequence, the Teflon^®^ insert is directly visible only in frames 75 and 76. This behavior reflects the large diffusivity contrast with the paperboard matrix combined with rapid heat redistribution, which limits the temporal persistence of the thermal signature. When PCT is applied to the original sequence, no meaningful EOFs associated with subsurface defects are observed, as global variance is dominated by environmental effects and slow thermal trends inherent to solar loading.

In contrast, RPCT applied to the original sequence reveals the synthetic dowel at the 9th EOF. The late appearance of this defect is consistent with its limited diffusivity contrast relative to paperboard and its high thermal inertia, which cause the associated thermal anomaly to be subtle and only separable at higher-order components, once dominant background contributions are removed.

A clearer differentiation between defects is achieved when the sequence is pre-processed using MdFIF. PCT applied to the MdFIF-filtered sequence highlights the rubber defect at the 26th EOF, confirming that thermal anomalies associated with small diffusivity contrasts can be detected only after the suppression of low-frequency trends and illumination artefacts. Finally, RPCT applied to the MdFIF-filtered sequence provides the most effective defect detection, revealing both the synthetic and rubber dowels at the 2nd, 8th, and 9th EOFs. This result demonstrates the synergistic role of MdFIF and RPCT: the pre-processing step enhances subtle, material-dependent thermal signatures, while RPCT efficiently separates sparse and localized anomalies from the low-rank background.

The numerical simulations further support this interpretation by providing a physically consistent description of the thermal field of evolution. The COMSOL model reproduces the modest surface temperature variations observed experimentally, with early-stage temperature differences on the order of 0.08 K and a maximum global variation of approximately 0.54 K during peak solar exposure, reflecting the strongly insulating nature of the book materials. Simulated temperature maps and gradient fields show that the influence of subsurface materials is primarily expressed through localized gradient variations rather than absolute temperature differences. Wool and Teflon^®^ exhibit the most pronounced responses, while rubber and synthetic dowels remain weakly expressed, in agreement with the experimental findings. The persistence of thermal signatures beneath decorticated areas and the enhanced visibility of Teflon^®^ in gradient maps further confirm the critical role of surface structure in modulating heat diffusion.

Overall, the hierarchy of defect appearance across raw thermograms, processed EOFs, and numerical simulation mirrors the magnitude of thermal diffusivity contrast between each insert and the paperboard matrix. This integrated thermophysical and statistical interpretation provides a robust and physically consistent framework for understanding defect detectability in solar loading thermography applied to layered paper-based cultural heritage objects.

It is also important to discuss the representativeness of the experimental defects adopted in this study. The dowels used in this study do not replicate the geometry or complexity of natural defects in ancient books, such as delamination, fiber degradation, or adhesive aging; their regular shape was chosen to provide a controlled ground truth for validating the thermographic workflow under solar loading. In this sense, the experimental validation relies on artificial defects with regular geometries, which provide a controlled ground truth for methodological validation but do not fully reproduce the irregular morphology or material heterogeneity of natural delaminations and degradation phenomena in historical books. Despite these differences, both artificial and natural defects affect local heat diffusion through contrasts in thermal diffusivity and resistance, so the fundamental thermal mechanism remains the same. This work should be regarded as a proof-of-concept and algorithm-validation study, with future research extending the approach to naturally degraded books and irregular defects.

A limitation of this study is the restricted sample size, limited to a single 19th-century book with fixed dimensions and structure (N = 1). While ancient books vary in binding, materials, thickness, conservation state, and period—factors that can influence heat diffusion—the aim here is to validate the diagnostic methodology under realistic, constrained conditions. Although this book is representative of layered paper-based heritage objects, variability across historical books may influence heat transfer and defect detectability, so broader validation is required. The approach exploits contrasts in thermal diffusivity and resistance within layered paper systems, a common feature across books, while variations in thickness or structure primarily affect detection sensitivity and timing rather than the underlying mechanism. Numerical modeling within the workflow allows adaptation of acquisition parameters to each artifact. This work should thus be viewed as a proof-of-concept, with future studies extending the method to a broader range of books and historical typologies.

Although the experimental dataset presented here was acquired under summer conditions in Harbin, the robustness of the proposed solar loading thermography workflow has been demonstrated in previous studies by the same research group across different geographic locations, seasons, and irradiance levels, including campaigns conducted in L’Aquila (Italy) under lower and more variable solar excitation [1,26]. In addition, several published applications of solar loading thermography on different categories of cultural heritage objects further support the portability of the approach and highlight the key role of adaptive signal processing in mitigating environmental variability [32].

Defects characterized by large diffusivity differences (wool and Teflon^®^) are clearly expressed in the numerical model and emerge experimentally only under favorable temporal and surface conditions, whereas defects with diffusivity values comparable to that of the host material (rubber and synthetic dowels) require advanced pre- and post-processing and appear only at higher-order EOFs. This integrated thermophysical and statistical interpretation provides a robust and physically consistent framework for understanding defect detectability in solar loading thermography applied to layered paper-based cultural heritage objects.

This work confirms that solar loading thermography can support reliable non-destructive diagnostics of movable book heritage, provided that advanced processing (MdFIF pre-filtering coupled with PCT/RPCT) is used to suppress environmental trends and enhance subtle, material-dependent signatures, while COMSOL modelling strengthens physical interpretability and extends applicability to valuable objects that cannot be directly exposed to solar loading.

A further practical limitation is the approximately 13-h acquisition time under natural solar loading, which represents a constraint for rapid in situ diagnostics and routine conservation workflows. While long-duration monitoring improves sensitivity to subtle thermal effects and low-contrast anomalies, it may limit field applicability. Future work will therefore focus on optimizing acquisition protocols to reduce monitoring time while preserving diagnostic reliability.

The numerical model aims to demonstrate that the polyvinyl acetate and paperboard layers, respectively, have a thermal diffusivity such that, coupled with the characteristics of the modest solar loading forcing, they generate a strong homogenization of the thermal field on the exposed surface shown by COMSOL Multiphysics^®^ and actually acquired by the thermal camera. Looking forward, the most effective use of these tools lies in a combined workflow where simulations guide acquisition planning (time windows/expected contrasts) and help validate or rank experimental EOF-based indications, enabling scalable monitoring protocols for other multilayered and low-contrast movable artworks, with reduced false positives and more targeted preventive conservation intervention.

Finally, given the unique and non-destructible nature of the historical sample, a quantitative analysis of defect burial depth limits was not in this study. Future research will address this by establishing quantitative detection limits using standardized industrial phantoms with variable defect depths [33,34,35].

## Figures and Tables

**Table 1:** Thermo-physical properties of the materials constituting the model.

Materials	Density [kg/m^3^]	Thermal Conductivity [W/mK]	Specific Heat at Constant Pressure [J/kgK]	Emissivity	Thermal Diffusivity [m^2^/s]
**O** **sb**	530	0.13	1266	0.94 [15]	1.94 × 10^−7^
**R** **ubber**	1100	0.15	1000	0.89 [16]	1.36 × 10^−7^
**W** **ool**	22 [17]	0.047	8.24	0.9 [18]	2.59 × 10^−4^
**T** **eflon**	2200	0.304	5.49	0.92 [19]	2.52 × 10^−5^
**P** **olyvinyl A** **cetate**	1190 [20]	0.25 [20]	1400 [20]	0.92 [20]	1.5 × 10^−7^
**C** **ellulose**	800	0.11	1750	0.55 [21]	7.86 × 10^−8^
**S** **ynthetic D** **owel**	2500 [17]	0.06	1500	0.9 [19]	1.6 × 10^−8^
**C** **otton**	448 [17]	0.065	4.76	0.77 [19]	3.05 × 10^−5^
**P** **aperboard**	900	0.072	1400	0.55 [21]	5.71 × 10^−8^

**Table 2:** Spot measurement of wind speed and wind direction acquired during the experimental campaign.

Date	UT Time	Wind Speed	Wind Direction
22 July 2025	24:00:00	5.69	217.13

## Data Availability

The data presented in this study are available on reasonable request from the corresponding author.

## Abbreviations

The following abbreviations are used in this manuscript:

MdFIF	Multidimensional Fast Iterative Filtering
MvFIF	Multivariate Fast Iterative Filtering
NDTs	Non-destructive techniques
PCT	Principal Component Thermography
RPCT	Robust Principal Component Thermography
SLT	Solar loading thermography
IRT	Infrared thermography
EOF	Empirical Orthogonal Function
LWIR	Long-wave infrared
GenAI	Generative artificial intelligence
